# Severe varicella in adult

**DOI:** 10.11604/pamj.2013.16.98.3591

**Published:** 2013-11-16

**Authors:** Loubna Benchat, Fatima Zahra Mernissi

**Affiliations:** 1 Department of Dermatology, CHU Hassan II, Fes, Maroc

**Keywords:** Varicella, exanthematous, rash

## Image in medicine

A 35-year-old man presented to the emergency with a 5-day history of fever, chest pain and exanthematous vesicular pruritic rash. His 10-year-old son had been diagnosed with chickenpox 2 weeks earlier. He had no history of chickenpox. On presentation, he was febrile 39.4°C, heart rate was 100 beats/min, and respiratory rate was 26 breaths/min. An extensive rash with macular and vesicles was noticed. The lesions were large, necrotic and hemorrhagic. The lesions were widespread affecting the face, trunk and the limbs. Laboratory studies showed thrombocytopenia and elevation of liver enzymes (AST, ALT). Chest X-ray revealed multinodular interstitial infiltrates in both lungs. The diagnosis of varicella with pneumonia and liver involvement was made. Varicella, the primary varicella-zoster virus infection, is predominantly a childhood disease in non-vaccinated populations. Although, it usually results in mild to moderate illness, serious complications can arise. Older age and a compromised immune system are the most important risk factors associated with severity of varicella disease. Pulmonary involvement, like in our patient, complicates between 5% and 15% of instances of adult chickenpox. Risk factors for progression to pneumonia include pregnancy, smoking, older age, chronic obstructive pulmonary disease and immune suppression. In patients who are immunocompromised, the mortality approaching 50% despite treatment. Most healthy adults have favourable outcomes. The other complications are secondary bacterial infections, Invasive infections (arthritis, osteomyelitis, necrotizing fasciitis, the central nervous system and ocular involvement). Treatment with intravenous antiviral is mandatory for patients with severe complications.

**Figure 1:**
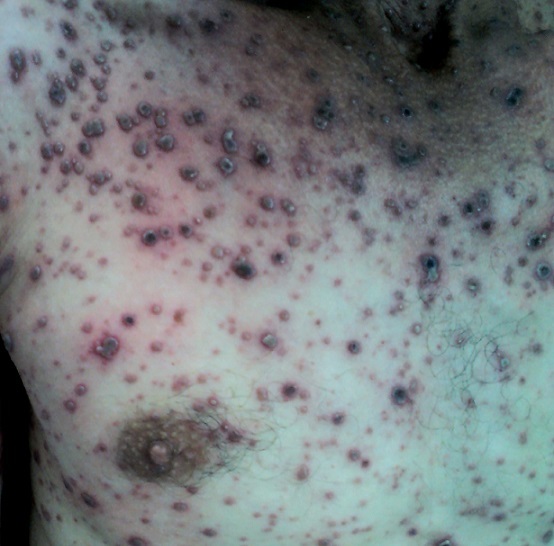
An extensive vesicular rash with large necrotic and hemorrhagic lesions

